# Population monitoring of trisomy 21: problems and approaches

**DOI:** 10.1186/s13039-023-00637-1

**Published:** 2023-05-14

**Authors:** Karl Sperling, Hagen Scherb, Heidemarie Neitzel

**Affiliations:** 1 Institute of Medical and Human Genetics, Charité-Universitaetsmedizin Berlin, Augustenburger Platz 1, 13353 Berlin, Germany; 2 Institute of Computational Biology, Helmholtz Zentrum München – German Research Center for Environmental Health, Neuherberg, Germany

**Keywords:** Down syndrome, Trisomy 21, Aneuploidy, Epidemiology, Risk factors, Preventive medicine, Sentinel phenotype

## Abstract

Trisomy 21 (Down syndrome) is the most common autosomal aneuploidy among newborns. About 90% result from meiotic nondisjunction during oogenesis, which occurs around conception, when also the most profound epigenetic modifications take place. Thus, maternal meiosis is an error prone process with an extreme sensitivity to endogenous factors, as exemplified by maternal age. This contrasts with the missing acceptance of causal exogenous factors. The proof of an environmental agent is a great challenge, both with respect to ascertainment bias, determination of time and dosage of exposure, as well as registration of the relevant individual health data affecting the birth prevalence. Based on a few exemplary epidemiological studies the feasibility of trisomy 21 monitoring is illustrated. In the nearer future the methodical premises will be clearly improved, both due to the establishment of electronic health registers and to the introduction of non-invasive prenatal tests. Down syndrome is a sentinel phenotype, presumably also with regard to other congenital anomalies. Thus, monitoring of trisomy 21 offers new chances for risk avoidance and preventive measures, but also for basic research concerning identification of relevant genomic variants involved in chromosomal nondisjunction.

## Background

Trisomy 21 (Down syndrome, DS) is the most common autosomal aneuploidy, even the most common genetic disorder among newborns. It is a sentinel phenotype, which can be easily recognized and cytogenetically verified. Trisomy 21 is registered in nearly all monitoring programs for congenital malformations as a paradigm for aneuploid mutations, e.g. in the *European Surveillance of Congenital Anomalies* (EUROCAT), a network of about 40 population-based registries, established in 1979 [1].

**Fig. 1:**
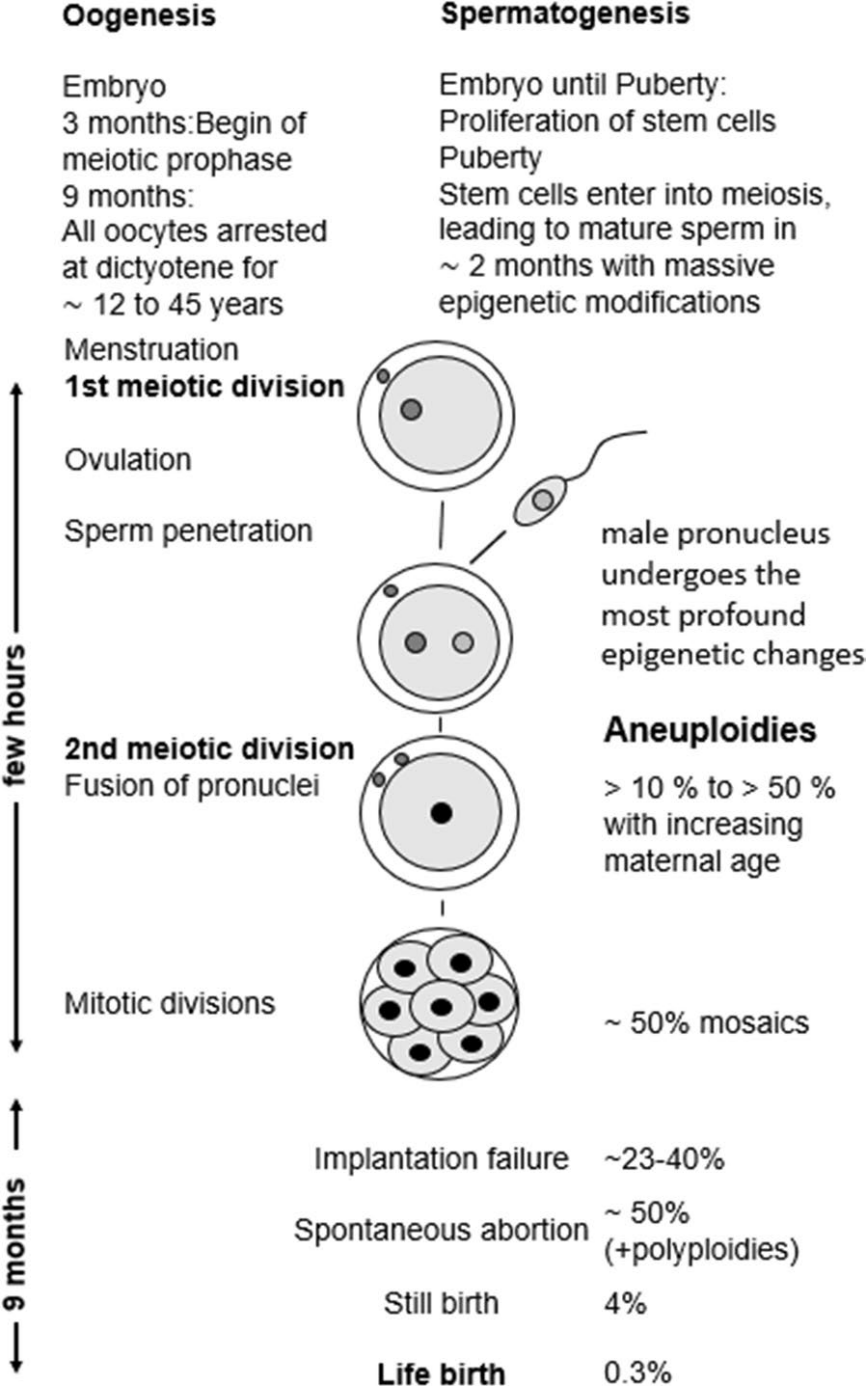
Human meiosis and rate of aneuploidies until birth

About 90% of trisomy 21 cases are due to maternal meiotic nondisjunction, whereby about 70% originate during the first meiotic division [M I] and about 20% during the second meiotic division (M II). A defective paternal meiosis is found for up to 8% of all cases. The risk of a DS birth increases over 40-fold between the ages of 20 and 45 in the absence of prenatal diagnosis and subsequent termination of pregnancy [2–5]. Thus, maternal age has the strongest effect on the rate of nondisjunction. An oocyte, which is ovulated by a 40-year-old woman, was arrested for about 40 years at the dictyotene stage of prophase I. During this long-time epigenetic modifications, resulting from a variety of environmental stressors, might affect MI nondisjunction, making their detection so elusive [6]. Sperm penetration of the oocyte is the trigger for MII. Consequently, the first and second meiotic divisions take place around conception (Fig. 1). Based on the molecular and temporal differences between maternal MI and MII it is assumed that also the risk factors for MI and MII nondisjunction errors are different [7, 8]. Nonetheless, maternal age is a dominant risk factor for both MI and MII nondisjunction [9]. However, the incidence of MI errors is high in the youngest mothers, lowest in the intermediate age group and increasing with advanced maternal age [10]. Moreover, the meiotic recombination patterns differ between both error types [8] and this is paralleled by variants in candidate genes coding for components of the synaptonemal complex [5]. Low socioeconomic status (SES) significantly increases the risk for DS and maternal MII nondisjunction [11–13]. Perhaps the lifetime exposures associated with low SES may generate some adverse effect at the time of oocyte maturation. In addition, smokeless chewing tobacco was associated with a higher risk for MII nondisjunction [14].

According to Coppedè [15] “the formation, development and survival up to birth of an individual with trisomy 21 should be viewed as a complex event involving environmentally induced epigenetic modifications, genetic factors, gene–environment and gene–nutrient interactions, and selection processes spanning over at least three different generations: the maternal grandmother, the mother and the developing trisomic individual”. In the present context it is important to note that numerous studies suggested that the DS risk is increased by environmental factors, but only few studies exist where the exposure is restricted to the time around conception, such as the exposure to short-lived radionuclides from the nuclear accident at Chernobyl or the exposure to the insecticide trichlorphon (see below). However, it is realistic to assume that environmental factors at the time of conception influence both MI and MII errors, but the latter to a relatively greater extent.

Given the molecular-cytogenetic differences and temporal separation between maternal MI and MII, it is not surprising that associated risk factors differ for MI and MII nondisjunction errors [7, 8]. Trisomy 21 is one of the few aneuploid conditions that survives to term, nonetheless about 50–80% conceptions are estimated to be lost during pregnancy [8]. Thus, about 90% of trisomy 21 cases occur around conception, when also the most profound epigenetic modifications take place [16–19]. Interestingly, most epidemiological studies report a male excess in DS, still a poorly understood phenomenon [20].

Altogether, the total aneuploidy rate in oocytes of young women is about 20–30% with an increase to more than 50% in women ≥ 40 years old [21]. Moreover, abnormal mitosis affects about 25% of the first three cleavage divisions [22, 23]. The frequency of aneuploidies in human preimplantation embryos varies between 50 and 80%, depending primarily on maternal age [24, 25]. Most of these embryos are lost during pregnancy. This incidence is more than an order of magnitude higher than in the mouse [26], which is one reason, why the extrapolation from mouse to man poses a fundamental problem with respect to the induction of nondisjunction.

From an evolutionary point of view, it seems a paradox that the high rate of aneuploidies in man, accompanied by reproductive failure, should represent a selective advantage. In fact, this is an adaptive mechanism to extend the interbirth interval from 9 months to 3 to 4 years, resulting in better overall survival rates. There is widespread agreement that humans are exceptional, birthing large-brained, helpless infants with a fetal pattern of brain growth for a year after birth. Thus, the human infant is more than any other primate dependent on maternal (parental) care. Lactational amenorrhea has evolved as one adaptive mechanism for spanning the birth interval preserving the health of mother and child [27, 28], another is the mostly unnoticed early pregnancy loss. This reduces maternal costs, which has been of particular importance in early human evolution [29, 30]. Thus, it is realistic to assume that aneuploidy is a natural occurrence in early human embryos [31, 32]. In other words, maternal meiosis is an error prone process that is sensitive to endogenous factors, as exemplified by maternal age, but also to recombination events in the maternal meiotic prophase as prerequisites for proper chromosome separation towards opposite poles [6, 33, 34]. Moreover, it is logical to assume that this process can also be affected by exogenous factors, especially by exposure around the time of conception.

Numerous studies suggested that trisomy 21 could be induced by environmental factors (recently reviewed by [5, 35–37]), but confirmatory evidence and generally accepted associations are missing so far. “The possibility that human aneuploidy may be induced by environmental factors such as smoking, drinking, oral contraceptive use and radiation exposure has been suggested by data from human studies over many decades …, but confirmatory evidence for these or any other agent has never emerged” [38]. High coffee consumption was even associated with an inverse effect, explained by reduction of DS conceptus viability [39]. In statements from 2022, addressed to the general public, *The National Down Syndrome Society* states “There is no definitive scientific research that indicates that Down syndrome is caused by environmental factors or the parents’ activities before or during pregnancy” [40] and the *Mayo Clinic* claims “There are no known behavioral or environmental factors that cause Down Syndrome” [41]. In this context the actual report of Reece & Hulse [42] that cannabinoid consume could increase the DS risk might be worth noting in the public discussion on cannabis legalization. Moreover, for epidemiological monitoring of DS the influence of socio-cultural and territorial variables is mostly unclear and only the impact of antenatal screening is evident [43].

In our opinion, the discrepancy between the generally accepted extreme sensitivity of trisomy 21 (and aneuploidy in general) to endogenous factors and the missing acceptance of causal exogenous factors is not biologically founded but mainly due to inherent problems of appropriate epidemiologic studies. The proof of an environmental agent is a great challenge, both with respect to ascertainment bias, including dosage and time of exposure but also with regard of the relevant co-factors affecting the birth prevalence, especially the strong maternal age effect and prenatal diagnostics with subsequent termination of pregnancy, as well as the high spontaneous loss of fetuses with DS. Moreover, it cannot be excluded that special groups of women exist with genetic disposition to different exposures. In addition, in case of only moderate exposure the population size might be too small and proper controls missing to detect clusters with a significant DS increase.

Clearly, there is an urgent need for epidemiological studies with respect to environmental hazards, including teratogens, affecting birth defects, both from a public health perspective of risk avoidance and primary prevention. In principle, monitoring of trisomy 21 as a sentinel phenotype could be a reliable and efficient approach and, as outlined below, also concerning its broader predictive value. The feasibility depends on standardized data collection and the reliability of ascertainment. In the nearer future these methodical premises will be clearly improved, both due to the establishment of electronic health registers and to the introduction of non-invasive prenatal tests.

The large number of efforts to establish an open data infrastructure for health data (e.g. initiated in 2020 by Gaia-X and accelerated by the COVID-19 pandemic) could be a breakthrough for a monitoring project with anonymous data and the identification of risk factors by pseudonymized documents. The use of electronic health records will provide data of unprecedented power, not only for individual clinical care and public health but also for biomedical research. Clearly, individual patient health data are sensitive and must be carefully safeguarded, whereas population health data are a highly valuable resource for basic and clinical research. Any *Health Information Technology* (HIT**)** system for health care must strive to balance these countervailing demands [44]. Moreover, a close collaboration with the relevant genetic support groups and their input into research could promote the translation of these studies towards clinical benefit [45].

With respect to ascertainment, the introduction of the non-invasive prenatal test (NIPD) for DS, based on fetal cell‐free DNA in mother´s blood from 10 weeks in pregnancy, offers a new approach. It has already shifted the national prenatal screening program of the Netherlands [46] and Belgium. NIPD is offered to all pregnant women in Belgium with an uptake above 75% [47]. This has enormous implications for DS diagnostics and monitoring, of course in due consideration of the guiding principles for the ethical, legal and social implications [48].

It is not the aim of this article to present a comprehensive review on the vast literature about possible exogenous risk factors of trisomy 21, but to illustrate the feasibility to identify these factors on a few instructive examples, also concerning its predictive value for other congenital disorders.

## Lessons from Down syndrome in Oman

An exemplary study on DS monitoring has been performed in the Sultanate of Oman from 2000 to 2004, both from an epidemiological and molecular point of view [49]. The Sultanate has 1.8 Mio nationals and a comprehensive health care system. More than 95% of approximately 40,000 births per year are examined by pediatricians, who prompt a cytogenetic analysis for confirmation of DS at the National Cytogenetic Service in Muscat. In about 90% of all cases the clinical diagnosis was confirmed cytogenetically within 6 months after birth. Ascertainment of DS can be considered almost complete. During this time, prenatal diagnostics and selective terminations of pregnancies did not play any role, birth control perhaps only a negligible role, an almost unique situation for DS monitoring. The mean number of pregnancies at birth for mothers of a child with DS was 8.7, range 1- 17 pregnancies. The mean age of DS mothers was 33.5 years and the mean age at first pregnancy 18.2 years.

From 2000 to 2004 the total number of live births was 200,157 and the number of DS cases 518. The average annual prevalence of free trisomy 21 was 1: 383 among newborns or 2.61 per 1,000 live births, which is one of the highest worldwide (see: International Clearinghouse for Birth Defects Monitoring Systems, Annual Report 2003).

Based on the maternal age-related risk for DS, the expected number of DS cases in Oman from 2000 to 2004 would be about 400, which is significantly less than the observed number of 518. The sex ratio with 1.31 was significantly different from that of the Omani controls with 1.06 (*P* = 0.002) and the unaffected sibs with 1.09.

**Fig. 2:**
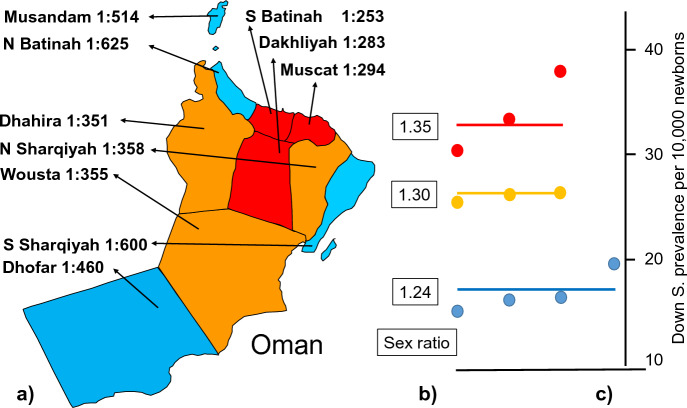
**a** Prevalence of Down syndrome (DS) in the 10 Omani regions from 2000 to 2004 per 10,000 newborns. Three different clusters could be identified: (red) highest, (dark yellow) middle, (blue) lowest DS birth prevalence/10,000 newborns. **b** DS sex ratio of the 3 cluster, **c** DS prevalence per 10,000 newborns for each region, assigned to the three clusters. The lines represent the mean value. The differences are highly significant (*p* < 0.0001; after [49], modified)

Interestingly, there were significant differences of the DS prevalence between the ten health regions of Oman. Altogether, three distinct clusters could be identified (Fig. 2). The DS cluster with the highest prevalence showed a ratio of 1: 304 (301 DS cases among 91,559 newborns), the middle cluster a ratio of 1: 381 (89 DS cases among 33,900 newborns), and the lowest cluster a ratio of 1: 584 (128 DS cases among 74,698 newborns). The mean maternal age of 34.7 years in high prevalence regions was not different from that of the low DS prevalence regions with 33.9 years. The cluster with the highest DS prevalence had also the highest DS sex ratio of 1.35, followed by 1.30 of the middle and 1.24 of the low DS prevalence cluster. The difference to the unaffected newborns is highly significant (*p* < 0.002).

**Fig. 3:**
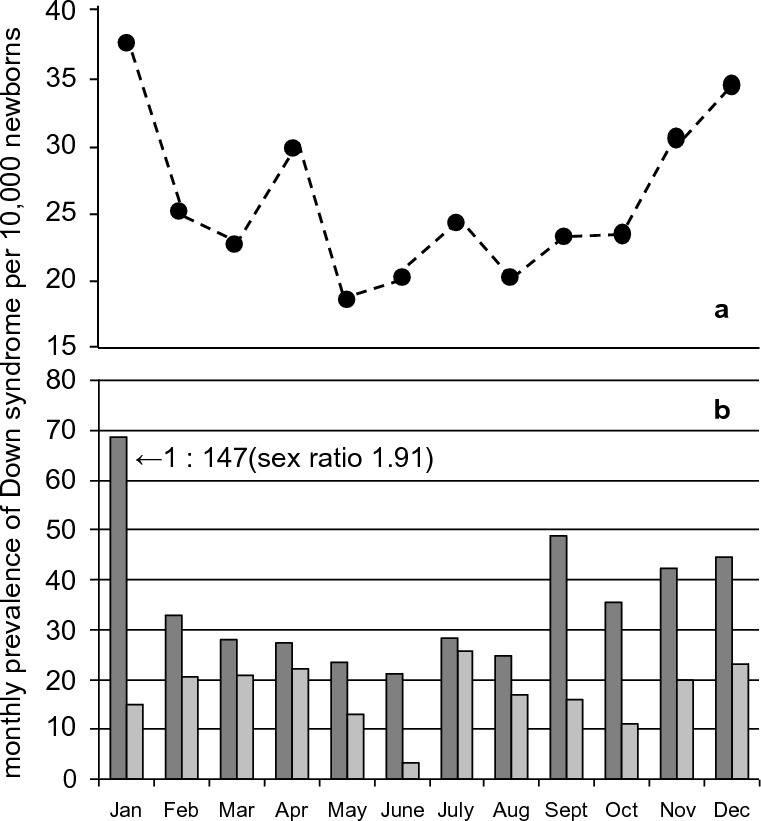
Monthly birth prevalence of Down syndrome (DS) in Oman from 2000 to 2004 (*N* = 518). a. All DS newborns. b. The regions with the highest DS prevalence South Batinah and Dakhliya (dark columns) and the regions with the lowest prevalence North Batinah, South Sharqiyah and Dhofar (light columns).The arrow points to January, the month with the highest prevalence (1 DS per 147 newborns; after [49], modified)

Interestingly, there was also a distinct monthly variation of the DS prevalence with the highest values in January, followed by December (Fig. 3a). Moreover, the cluster with the highest DS prevalence showed the most distinct seasonal variation with 1 DS per 147 newborns in January, while the cluster with the low DS prevalence has rather uniform monthly values. In addition, the highest sex ratio with 1.91 was also found in January in the cluster with the highest DS prevalence (Fig. 3b).

Moreover, the molecular-cytogenetic analysis of 338 cases showed that the cluster with the lowest DS prevalence had also the lowest number of maternal MII errors (18.8%), in contrast to the two other clusters with 37.3% (middle) and 31.5% (high DS prevalence). Altogether, 88.2% of all trisomies are of maternal, 8% of paternal meiotic nondisjunction, and 3.8% are due to mitotic nondisjunction.

The molecular analysis of short tandem repeat (STR) heteromorphisms of chromosomes 21 and Y and the sequence variation of the mitochondrial D-loop from 244 mothers showed that the rate of heterozygosity is almost identical with that published at the National Center for Biotechnology Information (NCBL). This indicates that the Omani population is genetically highly admixed, obviously due to extensive migration in ancient times. The rate of consanguinity of DS couples was not different from that of the general Omani population.

In addition, a case–control study on 90 cases and 90 matched controls (date of birth and region) was undertaken, covering amongst others socio-demographic data, information on menstrual history, individual and family health, exposure to X-rays, and occupational history. There were no obvious differences between cases and controls, e.g. approximately 70% of women in both groups were housewives and none of the women underwent special X-ray diagnostics or X-ray treatment.

The regions with the highest prevalence span the southern part of the coastal region at the Gulf of Oman, which is the most densely populated and principal agriculture area in Oman. A reporting bias as explanation for this January peak can be largely excluded as well as other confounders, such as maternal age, termination of pregnancy, or the rate of consanguinity. Consequently, an environmental cause is the most likely explanation for the December/January peak, almost confined to the cluster with the high DS prevalence, the high sex ratio, and the high rate of maternal MII nondisjunction. So far, no environmental factors have been identified that could explain this seasonal effect.

A possible candidate could be the intensive application of pesticides for agriculture protection [50, 51]. The increasing use of pesticides has been of particular concern in Oman, both in terms of human health and impacts on the environment. In 2006 the Pesticides Law regulated the application of pesticides, including the procedures for pesticide management and registration [52]. If the extensive use of pesticides in the past and the rate of DS in the high prevalence cluster are causally related, the restrictive use of pesticides should also decrease the DS rate. In this case, monitoring of trisomy 21 at this time could in retrospect through light on a health problem, apart from its reassuring effect.

Altogether, the significant regional and seasonal differences in DS prevalences, correlated with the sex ratio and MII errors and without relevant differences in the sociodemographic data, clearly point to an environmental causation.

## Lessons from Down syndrome near waste-disposal sites

The potential health risk of people living close to waste-disposal sites received great public attention, not least to the *Love Canal Tragedy* in the USA with hundreds of families even residing on contaminated land [53]. The exposure may be due to chemicals, like heavy metals, pesticides, carcinogens, and solvents released into the air, water and soil. This prompted the *European collaborative study of hazardous waste disposal in landfill sites and risk of congenital malformations* (EUROHAZCON) to perform two systematic studies, based on official registers of congenital anomalies in an area of 7 km radius around about 20 European landfill sites. The studies are based on the assumption that the exposure is higher in a proximate zone of 0–3 km than at 3–7 km distance from the waste landfill site. The cases with congenital anomalies (livebirths, stillbirths and termination of pregnancy with chromosomal anomalies) were classified according to the International Classification of Disease (ICD). In a first study only non-chromosomal congenital anomalies were included and as controls for each case two live births, born on the nearest following day in the 7 km area [54]. In a second study chromosomal congenital anomalies were recorded, amongst them 127 cases with Down syndrome, 38 living within 3 km of a waste-disposal site, and as controls 2,308 live births born in the same year [55].

There is a higher risk of trisomy 21 (DS) for families living close to the waste landfill sites and the increase is comparable with that for non-chromosomal anomalies (Table 1). The odds ratio for all non-chromosomal anomalies with 1.37 (1.33 adjusted for socioeconomic status and maternal age) is similar to the odds ratio for trisomy 21 with 1.33 (1.36 adjusted for maternal age).

**Table 1:** Odds ratios for Down´s syndrome [from 55] and selected congenital anomalies [from 54] among residents within 3 km of a hazardous-waste landfill site.

Congenital anomaly	N	Odds ratio (95%CI)
Down syndrome	38	1.33 (0.87–2.04); 1.36#
Neural-tube defects	130	1.86 (1.24–2.79)
Cleft palate	38	1.63 (0.77–3.41)
Malformations of cardiac septa	248	1.49 (1.09–2.04)
Renal abnormalities	75	1.30 (0.73–2.31)
Limb reduction defects	41	1.27 (0.61–2.62)
Multiple anomalies	84	1.21 (0.71–2.06)
Urinary tract anomalies	69	1.14 (0.62–2.11)

# adjusted for maternal age

If these results are not due to chance or a common bias, a direct relationship between residence near hazardous waste landfill sites and the increased risk for congenital anomalies remains as plausible explanation. The epidemiological approach, based on fixed thresholds in space to the place of delivery, is convincing. Clearly, the similarity in the risk estimates between cases with trisomy 21 and non-chromosomal anomalies does not point to a common underlying mechanism but illustrates the suitability of trisomy 21 as a sentinel phenotype. In this context, it is relevant that the global trade with hazardous waste from 2001 to 2019 in 28 countries is characterized by improper handling and still of high risk for human health [56].

## Lessons from Down syndrome after the Chernobyl reactor accident

After the Chernobyl accident in April 1986 the population of large parts of Europe was exposed to additional ionizing radiation (IR). While exposure to air-borne, short-lived radionuclides was limited to about two weeks, long term exposure, mainly due to caesium radioisotopes, lasted for many years. According to a global risk study on the health effect of the Chernobyl reactor accident "probably no adverse health effect will be manifest by epidemiological analysis" outside of the Chernobyl region [57]. In 2000 the United Nations Scientific Committee on the Effects of Atomic Radiation (UNSCEAR) stated: „Several studies on adverse pregnancy outcomes related to the Chernobyl accident have been performed …. So far, no increase in birth defects, congenital malformations, stillbirths, or premature births could be linked to radiation exposures caused by the accident “ [58]. This also refers to the increase of DS in West-Berlin, in Scotland and Sweden. According to the UNSCEAR report these observations were later challenged by a study published in 1997 [59]. With respect to DS the data of this study were collected from children´s hospitals in Bavaria. The inclusion criterion was treatment for congenital malformation within the first two years of life. Not all cases were cytogenetically confirmed and no prenatally diagnosed fetuses included. Thus, from an epidemiological point of view, DS recording is incomplete and in contrast to the ascertainment of DS in West-Berlin.

From an epidemiological point of view, the situation in West-Berlin, an “island” surrounded by the GDR, was unique, regarding the recording of trisomy 21 until the fall of the Wall in 1989. One university institute was responsible for genetic counseling and cytogenetic diagnostics, including prenatal diagnostics. More than 90% of all newborns with trisomy 21 were cytogenetically diagnosed already 10 days after birth. The age distribution of all mothers and of all pregnant women, who made use of prenatal diagnostics, was known [60]. 

During the 10 years from 1980 to 1989, the average monthly prevalence of trisomy 21 in West-Berlin was 2–3. In January 1987, 12 cases were observed, 10 newborns and 2 prenatally diagnosed fetuses. This increase was significant (*p* < 0.01) and occurred exactly 9 months after the Chernobyl reactor accident [60]. In an independent study in Germany in 1986, based on 28,773 prenatal diagnoses due to maternal age, the highest incidence of trisomy 21 concerns fetuses that were conceived in the same critical period as in Berlin and in the most heavily contaminated southern part of Germany (11 instead of the expected 4 cases). In the northern, almost unexposed part, the ratio between observed and expected cases was 6 to 5 [61].

**Fig. 4:**
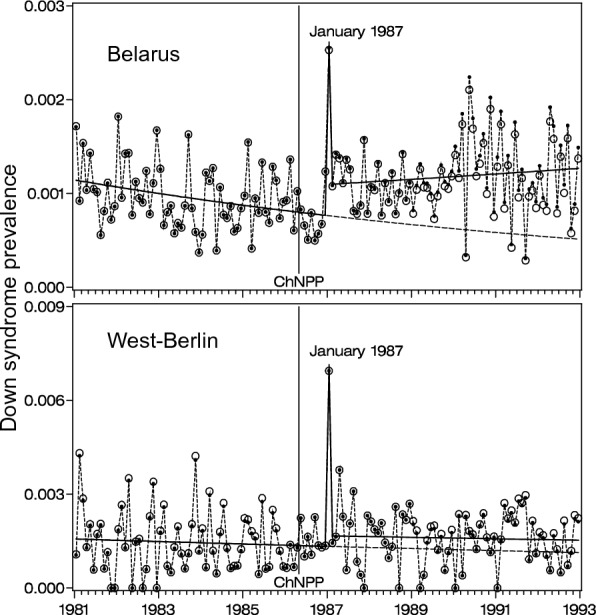
Raw (circle) and age adjusted (dot) monthly birth prevalence of Down syndrome from January 1981 to December 1992 in Belarus (*N* = 1,720,030; *n* = 1791) and West Berlin (*N* = 218,497; *n* = 237) based on a change point model allowing a decreasing trend with a peak in January 1987 and a jump, i.e. a level shift, for 1987 to 1992. Belarus: OR_peak_ *p* < 0.0001, OR_jump_ *p* = 0.0001; West Berlin: Or_peak_ *p* < 0.0001, OR_jump_*p* = 0.378 [from 64]

In January 1987 the monthly prevalence of Down syndrome among life births in Belarus also showed a highly significant peak (Fig. 4). Here again, ascertainment between 1981 and 1992 was high, prenatal diagnostics not performed and maternal age known [62].

The spatial distribution of children with trisomy 21, born in January 1987 in Belarus, followed the radioactive cloud’s passage within the first post-accident days. The same was true for Berlin. When the clouds from Chernobyl passed over, the weather was dry and sunny and the only exposure of the population was due to the inhalation of short-lived radionuclides, in particular iodine 131 (physical half-life 8 days, biological half-life even less), for most of the DS mothers exactly at the time of conception. Since Belarus and Berlin are countries with a high prevalence of iodine deficiency, the uptake of radioactive iodine was higher than in most other European countries and could be about 0.05 mSv during the critical two weeks.

In both areas, the most important confounders, such as maternal age distribution, number of prenatal diagnostics, and completeness of ascertainment could be excluded. Moreover, also the generally recognized criteria of Bradford Hill [63] are fulfilled: strength of the association, specificity, relationship in time, consistency, biological gradient (dose–effect-relationship), experimental evidence, biological plausibility, and reasoning by analogy.

In addition, a long-term effect was also observed in the first post-accident years, not only in Belarus but also in several other European countries or regions, such as Bavaria in Germany, the Lothian region of Scotland, North-West England, Hungary, and Sweden. This effect has been explained by exposure, especially to Caesium-137 (physical half-live 30 years), which reached its maximal uptake about 1 year after the Chernobyl accident [64].

The January 1987 peaks of DS in Berlin and Belarus represent a strong association, any relevant bias or confounder should be easily identifiable. The long-term effect is less distinct, but characterized by its uniform beginning in 1987**,** making an artefact rather unlikely. This long-term effect may be even more important than the January peak from a public health point of view. Thus, the Chernobyl effect is exceptional, both with respect of its specificity in time and reproducibility, leading to the assumption of a causal relationship between low-dose irradiation and meiotic nondisjunction in man. Actually, there is good evidence for an inverse dose–effect relationship according to which also other types of hereditary defects, cancers included, could be increased after low dose of irradiation [65].

Moreover, in West-Berlin in January 1987 the sex ratio among the 10 DS newborns was 8 males to 2 females. Interestingly, in the years following the Chernobyl accident, a significant increase in the sex ratio, confined to the exposed European countries and Cuba, has been documented, which is due to a decrease of female newborns [66–69]. In Bavaria, this increase of male births was already observed in January 1987, whereby this effect was higher in the more heavily exposed southern part (6.9%) than in the northern part (3.5%) [70]. The critical stage for this shift is the time around conception. The increase in the sex ratio after exposure to low dose of ionizing radiation has been explained by an epigenetic effect, defective X-inactivation, resulting in a loss of female embryos [71]. The phenomenon of male excess in DS has been explained by co-orientation of chromosomes Y and 21 in male meiosis, a greater accessibility of Y-sperm to a disomic zygote or to post-fertilization events leading to selection against females, as in case of defective X-inactivation [20].

It should be added that also other health effects after in utero exposure by the Chernobyl fallout had been documented in Germany, e.g. an increase of stillbirths and congenital malformations (Table 2). Especially in Bavaria, a dose-dependent significant increase of congenital malformations, beginning in 1987, was documented [72].

**Table 2:** Health effects after in utero exposure by the Chernobyl fallout in the Federal Republic of Germany (FRG) and the Democratic Republic of Germany (GDR) (from [96] and references therein)

Health effect	Region
Perinatal deaths, stillbirths	Total (FRG + GDR)
Infant mortality	Southern Germany
Cleft lip and/or palate	GDR, Bavaria
Reduced birth rate	Bavaria

With respect to ascertainment of trisomy 21 the “island” situation of West Berlin was exceptional. Diagnostics of trisomy 21 was part of the regular medical service and supported by the excellent official health statistics provided by the Senate of West Berlin. It can, perhaps, serve as a model for monitoring of trisomy 21, nearly without any additional costs. In addition, it is one of the few cases where the impact of the environmental factor was limited to the time of conception.

## Lessons from a Down syndrome cluster in Hungary

In 1991/1992 in Rinya, a small Hungarian village with 456 inhabitants, 11 of 15 newborn had congenital anomalies, among them 4 with Down syndrome (2 were monozygotic twins). Of 61 children born between 1980 and 1988 only 3 had malformations. The 10 children born in 1991/92 were healthy [73].

Apart from the 4 cases with Down syndrome, the other probands had different anomalies, which could be assigned to critical embryonal periods (Table 3). A familial genetic disposition, consanguinity, or a chance event could be virtually excluded. The most likely explanation was the impact of a teratogenic factor.

**Table 3:** Selection of children born in Rinya in 1989 and 1990; # Fish consumption: +++ = often, in critical period certainly; ++= often, in critical period probably; + = occasionally, in critical period probable; (+) = questionable.

Congenital anomaly	Date of conception	Critical period	Fish consumption #
Ventricular septal defect + pulmonary atresia	11.05.1988	Days 30–38	+++
Down syndrome	06.04.1989	conception	+*
Stenosis of left bronchus (70%)	20.04.1989	Days 34–44	+
Anal atresia	20.11.1989	Days 36–41	+
Choanal atresia	03.02.1990	Days 44–51	+
Cleft lip, left	20.02.1990	Days 35–50	++
Down syndrome—MZ twins	16.04.1990	conception	+++
Down syndrome—DZ twins	13.04.1990	conception	(+) *
Robin sequence	13.04.1990	Days 49–56	(+)

*RFL polymorphism points to nondisjunction in MII; RFL polymorphism not informative [from 73]

The exemplary population-based “Hungarian Congenital Abnormality Registry” proved that the cluster was confined to this small village. The following environmental investigation disclosed the excessive use of trichlorfon, an organophosphorus insecticide for eradication of parasites, at local fish farms. Several pregnant women had consumed contaminated fish in the critical period for the congenital anomalies observed, the mothers of the children with DS around conception. The molecular analysis showed an MII error in two cases, one was not informative (Table 3). The concordance between the time of exposure and the most sensitive time for the induction of the different malformations was another argument for a causal relationship. When the trichlorfon treatment of the fish was banned, the cluster ceased.

Clearly, this was a retrospective study, an observational approach after the cluster has ended. Nonetheless, the concordance between the time of exposure and the most sensitive time for the induction of the particular malformation not only points to a causal relationship but illustrates that monitoring of trisomy 21 might also be relevant for the understanding of teratogenic effects and applicable to small cohorts.

## Concept for population monitoring of Down syndrome

As outlined above, the high rate of maternal meiotic nondisjunction in man, leading to trisomy 21, represents an error prone process. Most cases occur around conception, when also the most profound epigenetic modifications take place. Thus, from an epidemiological point of view, surveillance of trisomy 21 offers a unique chance for monitoring this sensitive process and for identification of endogenous and exogenous risk factors [74].

For reasons of efficiency (time and money), it is generally desirable to use existing databases, which should include the most important confounders, such as parental age at conception, offspring age and sex at diagnosis, as well as population-based data, such as usage of prenatal screening and termination of pregnancy in case of DS, as well as place of residence.

Concerning the registration of the relevant individual and population health data, the guiding principles developed for the application of genomics to human health and disease can serve as a model [48, 75–77]. While individual health data are sensitive and should be safeguarded, population health data are a most important resource for research. A balance has to be found between these countervailing demands, both on a national and international basis [58, 78]. The collection, storage, and processing of sensitive health data is the aim of the European digital GAIA-X initiative. It should also enable the connection to other data platforms to further research and health domains (https://www.data-infrastructure.eu/GAIAX/Redaktion/EN/Artikel/UseCases/smart-health-connect.html). A vision for the future is the development of differential privacy technology within cloud-sharing communities protecting the privacy of users and resolving the data-sharing problem [79].

The recent initiatives to help enable and foster data sharing practices for pediatric research and translate these into practice, also with respect to consent clauses, are reviewed by Patrinos et al. [80]. Today, Denmark has perhaps one of the most effective health systems, based on the establishment of a national e-health portal, sundhed.dk, providing patient-oriented digital services. The Danish Health Data Authority’s Research Services supports health research in Denmark by providing access to register-based health data via a secure research platform [81]. Also the Western Australian Data Linkage System (WADLS), instigated in 1995, can serve as a model to link local health and welfare data sets, genealogical links, and spatial references for aetiologic research, disease surveillance and methodic research [82]. This register combines information related to antenatal and perinatal factors, contains all registered births, and provides information related to parental age. In addition, it provides information on all registered deaths and causes of mortality. Each individual congenital anomaly is coded using the International Classification of Diseases system [83]. The WALDS is used intensively and receives a high level of social acceptance, which is based primarily on the involvement of a wide variety of social groups, including patient self-supporting groups. Clearly, these initiatives, such as WALDS, can also serve as model for a monitoring project with DS data and the identification of risk factors.

Concerning the reliability of DS ascertainment, the most important aspect is that the DS birth prevalence is counterbalanced by the number of pregnancies that are terminated due to the availability of prenatal screening. In 2011, sequencing of cell-free DNA in maternal serum as a sensitive, non-invasive screening technique (NIPD) has been developed, which can be performed already at 10 weeks of gestation. Until 2020 about 10 million tests have been performed [5]. The prediction rate of trisomy 21 is superior to first trimester combined screening based on maternal age, fetal nuchal translucency thickness (NT) and serum markers [84]. The positive predictive value for NIPD was significantly higher than that for standard screening (45.5% vs. 4.2%, [85]; (80.9% vs. 3.4%, [86]. Thus, the positive predictive value for trisomy 21 is significantly improved, explaining its broad application worldwide. Moreover, the average fetal loss rate between the time of NIPD/ chorionic villus sampling and term is more than 30% [87]. Thus, the monitoring efficiency is also increased in comparison to newborn screening. NIPD, however, is not a diagnosis and needs confirmation by invasive diagnostic testing [88]. If, in the future, this diagnosis is no longer carried out cytogenetically but by whole genome sequencing, this would open up entirely new possibilities for the identification of genetic risk factors.

The implementation of NIPD differs between market-based and state-sponsored health systems [89]. Today, this test is already offered in Belgium and the Netherlands to all pregnant women [46, 47]. Here is not the place to discuss the ethical/cultural implications of prenatal diagnostics and reproductive autonomy, both with respect of termination of a DS fetus but also concerning the benefits to initiate early treatment [5]. Clearly, for population monitoring of trisomy 21 NIPD offers completely new aspects with respect to population-wide ascertainment in early pregnancy. Moreover, direct haplotyping has been successfully applied for NIPD of monogenic disorders [90–92] including triplet-repeat expansion diseases [93]. Based on the significant progress of NIPD in the past few years, it is realistic to assume that in the future haplotyping of aneuploidies is possible as well as differentiation between MI and MII errors.

For population monitoring of DS an ecological study is applicable as generally used in public health research. The results should be evaluated by the Bradford Hill criteria in case of a causal suspicion. A case–control study, however, has greater power to identify specific (environmental) risk factors. Due to its retrospective nature, case–control studies are subject to recall bias, but are inexpensive, efficient, and especially suitable for rare diseases [94]. The Hungarian study in 1991–1992 is a paradigm of this approach [73]. Based on the Hungarian Congenital Abnormality Registry, the history of the mothers of these cases could be obtained with little extra effort. In this case the “controls” were the 60 offspring born before and after the critical period.

Generally, in case–control studies the cases are the families with an affected child and the controls those with an unaffected child. Based on personal interviews with a structured questionnaire of 50 mothers, who gave birth to a child with trisomy 21, and 272 controls the highest odds ratios were found for thyroid scan and the investigations of the pelvis and the abdomen. About 90% of cases of the study population and contacted controls participated, underlining the feasibility of this approach [74]. In an extensive comparison of odds ratios using affected and unaffected controls there was no evidence that differential recall of exposure has an important implication in case–control studies of birth defects [95].

If the health data are routinely collected as in Western Australia, case control studies on trisomy 21 can be performed without any extra efforts and, apart from the monitoring aspect, pave the way for applied research concerning causally relevant environmental factors but also with respect to basic research related to relevant genomic variants and, perhaps, help to explain the increase of the sex ratio.

## Conclusion

Trisomy 21 is a singular resource to understand meiotic nondisjunction and its endo- and exogenous risk factors in humans, as it is one of the few aneuploid conditions that is recorded at early embryogenesis and can, in principle, survive to term. Thus, monitoring of trisomy 21 as sentinel phenotype for congenital anomalies in general, offers new chances for preventive measures. The epidemiological prerequisites with respect to completeness of ascertainment, consideration of the relevant confounders, and application of appropriate statistical approaches are still challenges. Nonetheless, the advents in molecular genetics including non-invasive prenatal screening and the global initiatives for the implementation of health data registers offer most promising chances for basic medical research.

## Data Availability

Data sharing is not applicable to this article as no datasets were generated or analyzed during the current study.

## Abbreviations

DS: Down syndrome
DZ twins: Dizygotic twins
EUROCAT: European Surveillance of Congenital Anomalies
FRG: Federal Republic of Germany
GDR: German Democratic Republic
HIT: Health Information Technology
ICT: International Classification of Disease
IR: Ionizing radiation
MI: First meiotic division
MII: Second meiotic division
MZ twins: Monozygotic twins
NCBI: National Center for Biotechnology Information
NIPT: Non-invasive prenatal test
RFL polymorphism: Restriction length polymorphism
STR: Short tandem repeats
WADLS: Western Australian Data Linkage System
